# Facilitated Alveolar Ridge Expansion Using Osseodensification: Achieving Implant Stability and Tissue Remodeling Without Grafting

**DOI:** 10.7759/cureus.80202

**Published:** 2025-03-07

**Authors:** Chen-Che Hung, Fernando Rojas-Vizcaya

**Affiliations:** 1 Periodontology, Mediterranean Prosthodontic Institute, Castellon, ESP; 2 Dentistry, Hung Kuntsung's Dental Clinic, Kaohsiung City, TWN; 3 Prosthodontics, University of North Carolina School of Dentistry, Chapel Hill, USA; 4 Implant Prosthodontics, Mediterranean Prosthodontic Institute, Castellon, ESP

**Keywords:** alveolar ridge expansion, buccal bone thickness (bbt), buccal bone wall, graftless implantology, implant stability, osseodensification

## Abstract

Proper placement of dental implant restorations and their longevity are critical factors to consider before starting the treatment. It is essential for the implant to achieve primary stability upon insertion and undergo osseointegration during and after the healing process. Implant placement should follow prosthetically driven planning to ensure optimal outcomes. In cases of a narrow alveolar ridge, achieving mechanical stability poses a challenge while also preserving the buccal bone wall. A novel technique, osseodensification, has been introduced to address these issues by facilitating implant placement within a densified socket while also preserving the buccal bone wall through its ridge expansion techniques.

This case presentation highlights using osseodensification for ridge expansion in low-density alveolar ridges. This technique preserves and increases the volume of the buccal bone wall while also reshaping the soft tissue above.

## Introduction

Over the decades, dental implant restoration has become a promising and reliable treatment for restoring missing teeth in both functional and aesthetic aspects [1]. Placing the implant in the correct position and achieving implant osseointegration while preserving enough bone tissue and maintaining the peri-implant tissue is crucial for achieving optimal long-term outcomes [1-3]. To establish a better osseointegration result, implant stability plays a significant role in the healing process through alveolar bone remodeling and increases direct bone-to-implant contact (BIC) [4,5]. Monje et al. [5] concluded that primary stability leads to more sufficient achievements than secondary stability. High mechanical engagement attained a higher implant survival rate.

Biological and esthetical functions should be accomplished while placing the implants. An adequate residual buccal bone wall must be preserved to ensure that the implant is placed correctly within the alveolar housing, preventing possible clinical implications such as peri-implant tissue resorption [6-8]. A single implant placement following the prosthetically driven 3A-2B rule guideline, positioning the implant 3 mm from the cervical contour of the planned crown approaching appropriate biological width and preserving approximately 1.8 to 2 mm of the buccal bone wall, is widely conducted in clinical studies [1,6,8]. The residual buccal wall tends to sustain the vestibular volume of the soft tissue, which favors the purpose of esthetics [8-10]. A thick buccal bone wall (greater than 1.5 mm) may reduce the risk of gingival margin recession and implant dehiscence, as well as facilitate tissue creeping and stability over time [10].

For narrow alveolar ridges, various ridge expansion techniques are proposed, with or without guided bone regeneration, to augment the implant site and achieve the most satisfactory results [9,11-13]. A novel technique, osseodensification (OD), is introduced to densify the alveolar bone during the osteotomy using specifically designed burs in a counterclockwise rotation [12-14]. The reverse non-subtractive drilling technique uses hydraulic compression to direct irrigation to compress against the osseous tissue of the lateral walls of the preparation. A series of bouncing-pumping motions of the burs are performed, allowing OD to compress and release the force inside the osteotomy [12-16]. This technique effectively compresses the autogenous graft and increases the density of the osteotomy socket, enabling the implant to achieve greater insertion torque and enhanced BIC, promising a higher implant stability quotient (ISQ) and resonance frequency analysis (RFA) [12,14-18]. Some authors have claimed that this technique can expand the alveolar ridge under outward strain, resulting in ridge plastic deformation, increasing buccal bone wall thickness, and allowing for simultaneous implant placement [12,13,19,20]. Research comparing OD drilling to conventional osteotomes or ridge expanders in ridge expansion has been published, highlighting the simplicity and dependable results of OD in clinical trials [19,20].

Ridge expansion through the OD method results in increased alveolar bone density, which enhances implant stability while simultaneously expanding the ridge in specific preparation techniques [13,19]. The specially designed “Densah” burs kit by Versah LLC (Jackson, MI, USA) is a set of taper-shaped burs that feature multiple flutes and lands, capable of generating hydraulic pressure within the osteotomy. The hydraulic pressure compresses the autogenous bone particulates, increasing bone density and expanding the alveolar ridge due to the viscoelastic properties of the bone tissue [13,14,19].

The case report presented a single posterior implant restoration following the guideline of the 3A-2B rule using OD ridge expansion protocol at the right mandibular region, showing the result of increasing buccal bone wall thickness and soft tissue recontouring after the treatment.

## Case presentation

**Figure 1:**
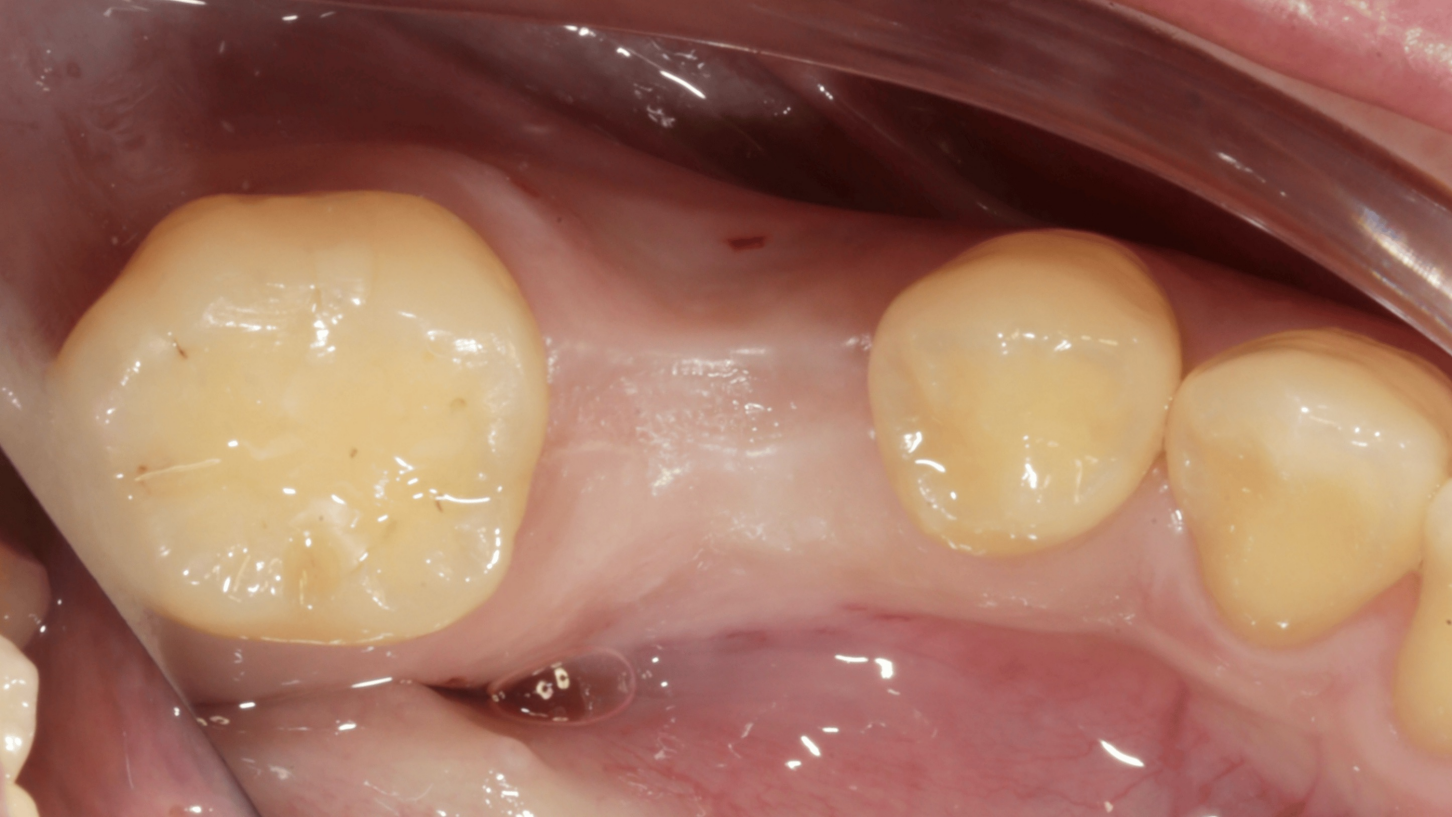
An edentulous mandibular right second premolar with a shallow cavity resulting from alveolar ridge resorption.

A 37-year-old female patient visited Hung Kuntsung’s Dental Clinic to undergo implant restoration for her mandibular right second premolar, which had been extracted over two years prior due to a fracture. No clinical symptoms or discomfort were observed within the oral cavity. Intraoral photographs, a cone beam computed tomography (CBCT) scan (RyanScan Studio, Ray Co., Ltd, Seongnam, South Korea), and model casts were collected to document and analyze the situation. The buccal surface of the alveolar ridge was recessed, which showed a shallow concavity (Figure 1).

**Figure 2:**
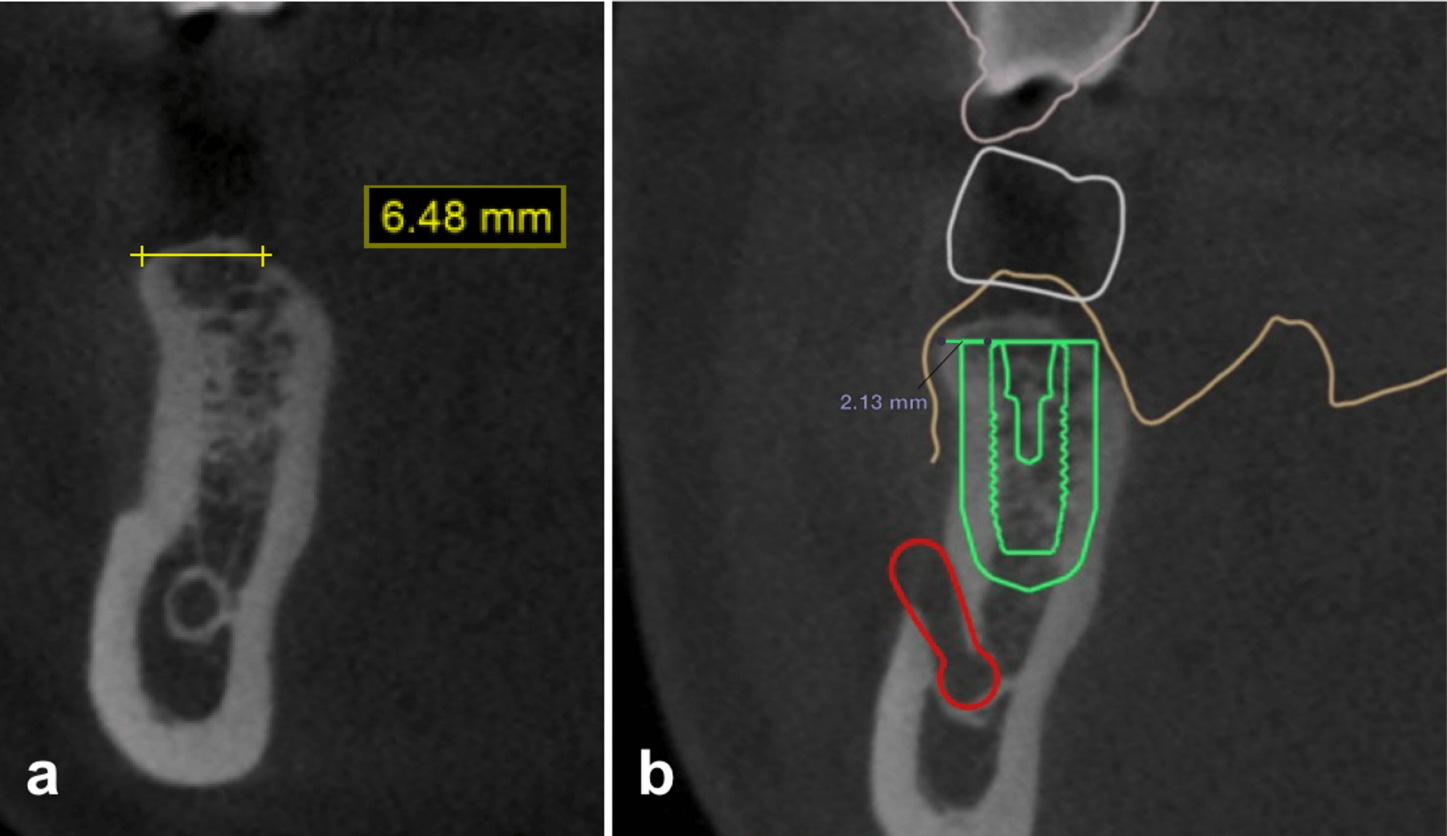
CBCT view of the initial alveolar ridge (a) and treatment planning using the 3A-2B rule (b). CBCT, cone beam computed tomography

The sagittal view of the CBCT (Blue Sky Plan, Blue Sky Bio LLC, Libertyville, IL, USA) indicated low density in the alveolar ridge with a prominent cortical bone outline. The residual alveolar ridge width was measured as 6.48 mm with a lingual slope (Figure 2a). Crown and implant positions were planned using digital implant treatment planning software (Implant Studio, 3Shape, Copenhagen, Denmark). Around 2.13 mm of the buccal wall was planned to be preserved after the osteotomy, and a 2 mm safety border of the implant was marked in the treatment planning (Figure 2b).

**Figure 3:**
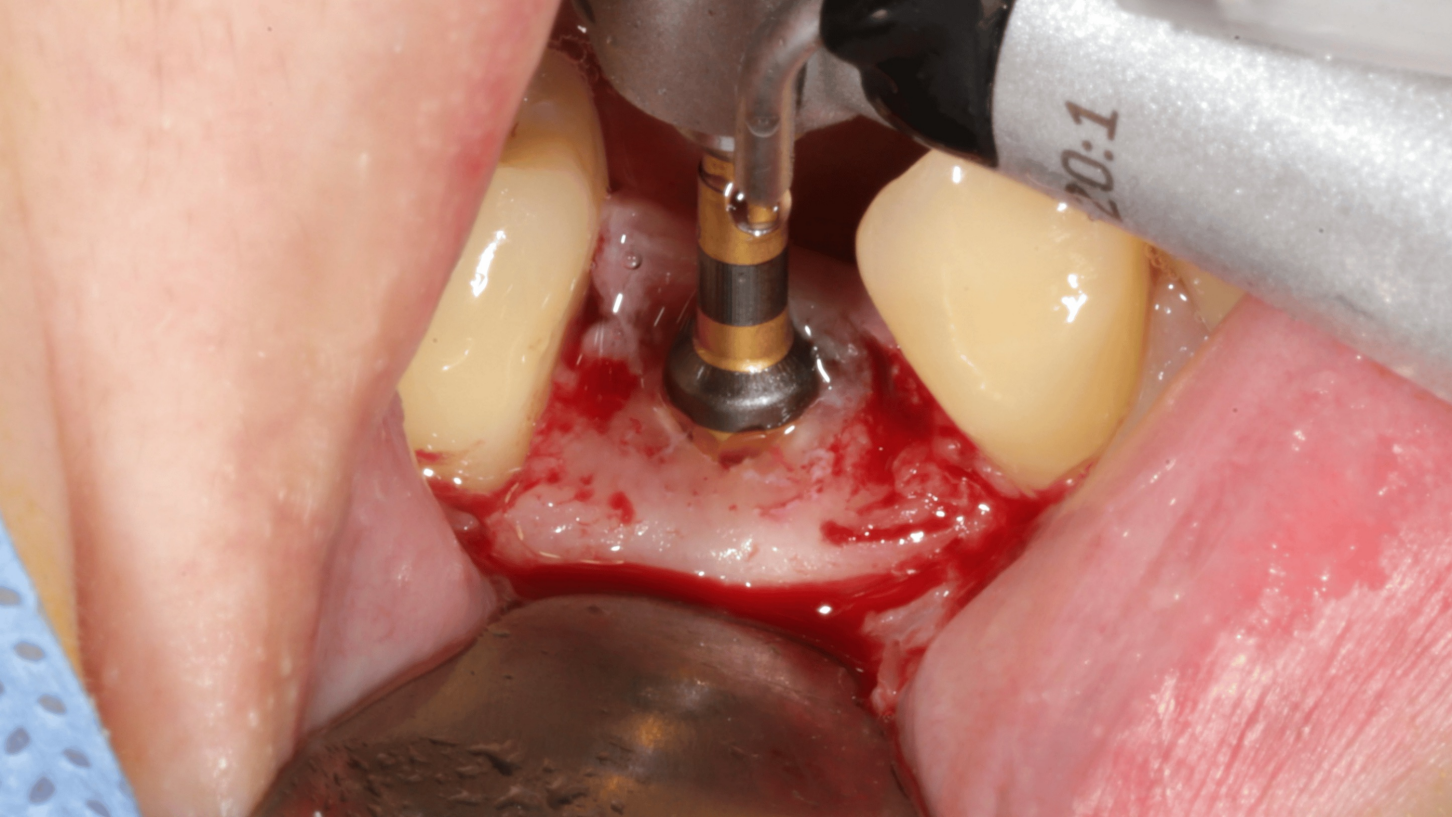
Osseodensification using Densah burs.

**Figure 4:**
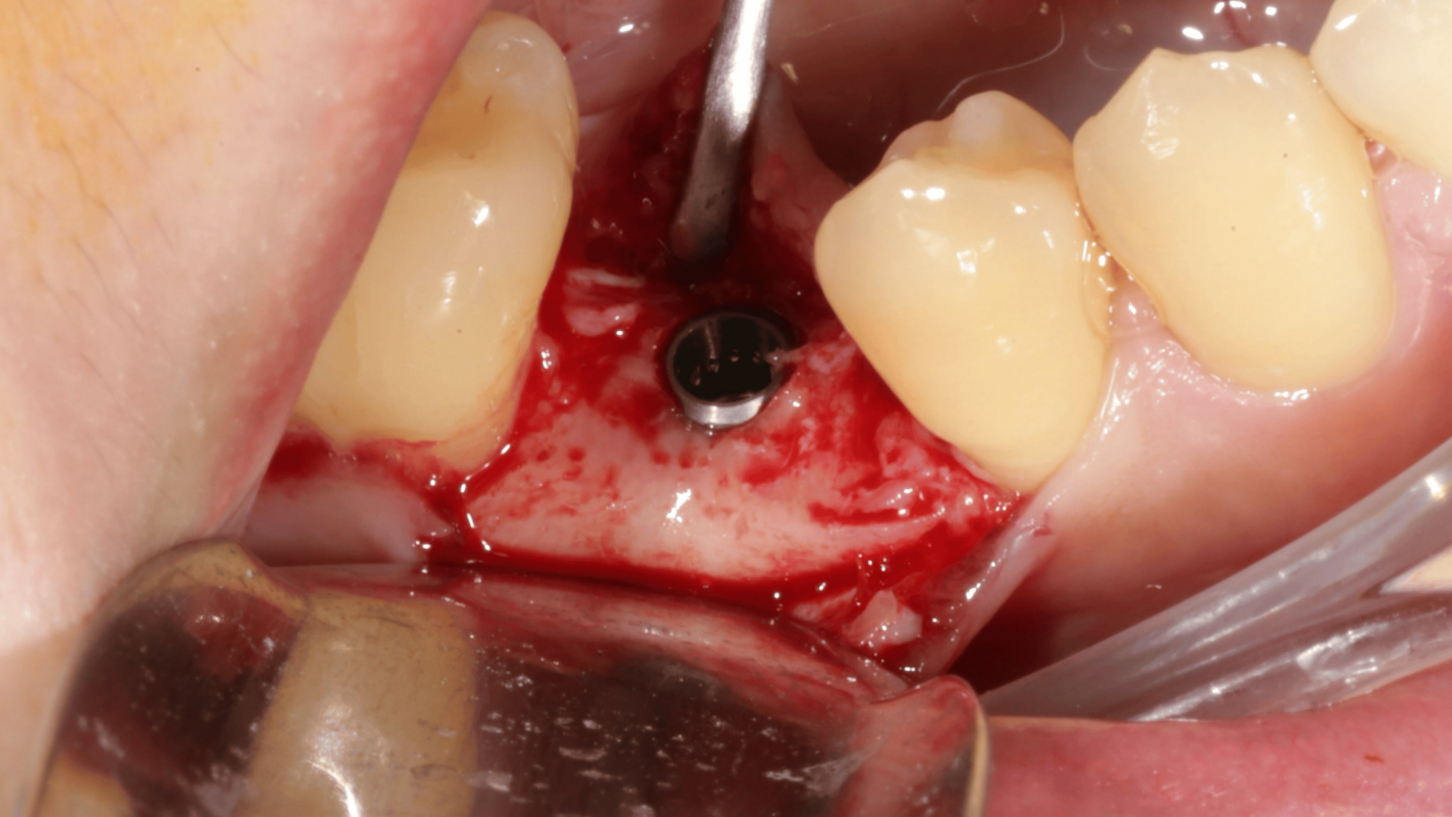
Implant placement at the bone level.

A thermoplastic surgical stent (EZ Stent, AD Surgical, Sunnyvale, CA, USA) was first fabricated from the model cast based on measurements recorded by the software and subsequently tested intraorally. Following the incision, the flap was retracted buccally without any vertical incisions, and the stent was utilized for 2.0 mm pilot drilling. The implant osteotomy was performed using the Densah Bur (Short Densah Bur, Versah LLC). The OD counterclockwise (CCW) drilling followed diameters of 2.3 mm, 3.3 mm, and 3.5 mm (Figure 3) at the speed of 1,200 rpm. A 4.0 x 11 mm implant (OsseoSpeed TX, ASTRA TECH, Dentsply Sirona, Charlotte, NC, USA) was inserted at 35 N torque and positioned at the bone level (Figure 4). A 4.5 x 4 mm healing abutment (ASTRA TECH, Dentsply Sirona) was placed to promote soft tissue healing. The flap was sutured with 4-0 nylon (Dafilon, B. Braun, Melsungen, Germany) using vertical mattress suturing to close the wound. Postoperative medication was prescribed, including mefenamic acid 250 mg and amoxicillin 500 mg, to be taken every six hours for the following week. The patient was also advised to use chlorhexidine mouthwash twice daily during this period.

**Figure 5:**
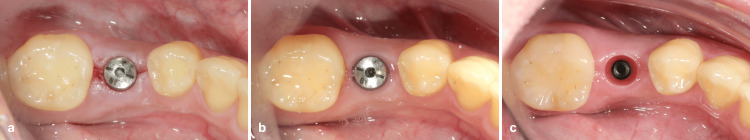
Follow-up in one week (a), three weeks (b), and six months (c).

**Figure 6:**
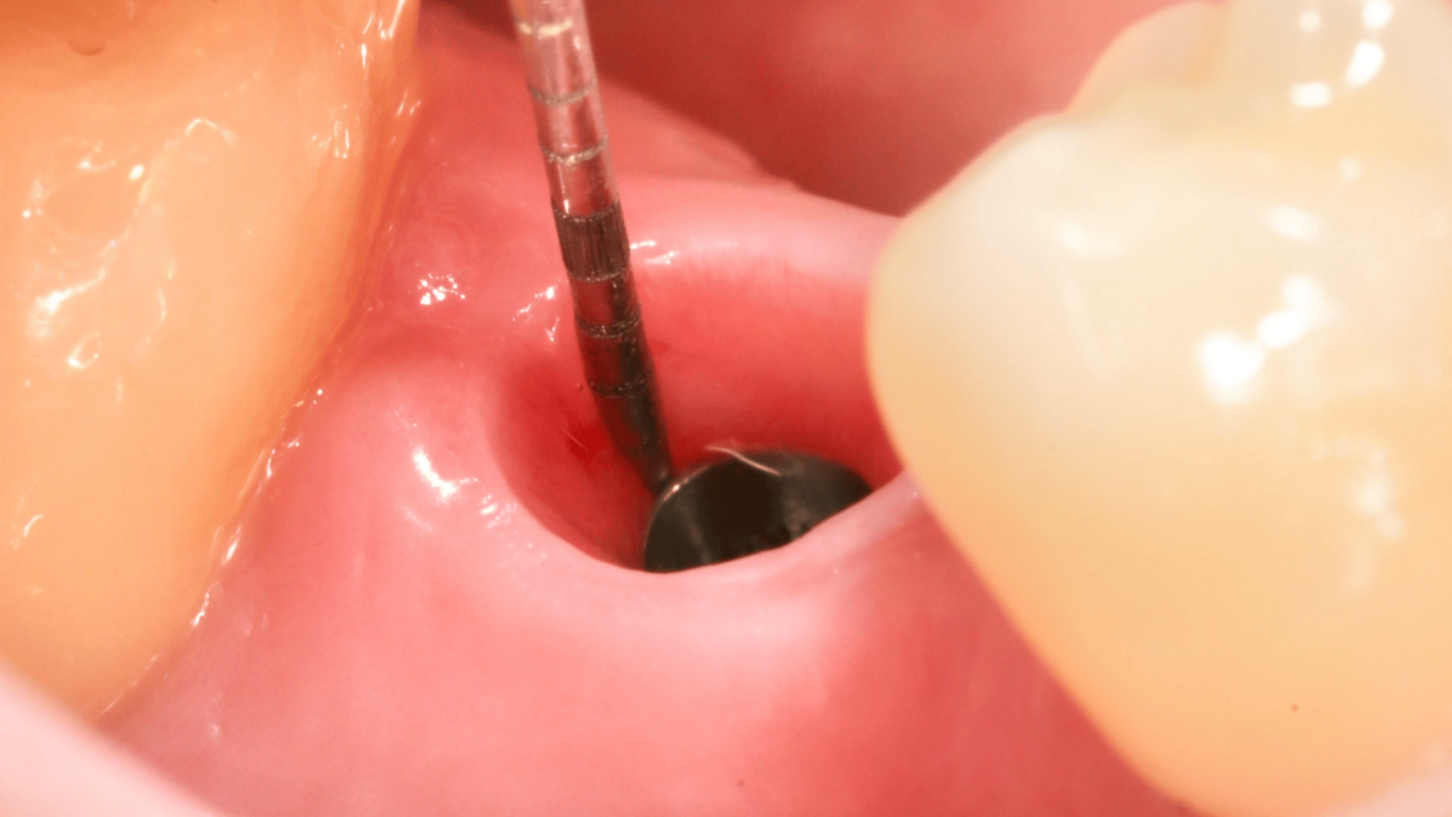
Peri-implant tissue after six months of healing.

The sutures were removed after one week, and the implant site was regularly monitored for three weeks and six months (Figure 5). The buccal surface of the gingiva has shown inflated and reshaped tissue throughout the healing periods. The peri-implant tissue was maintained and recorded (Figure 6).

**Figure 7:**
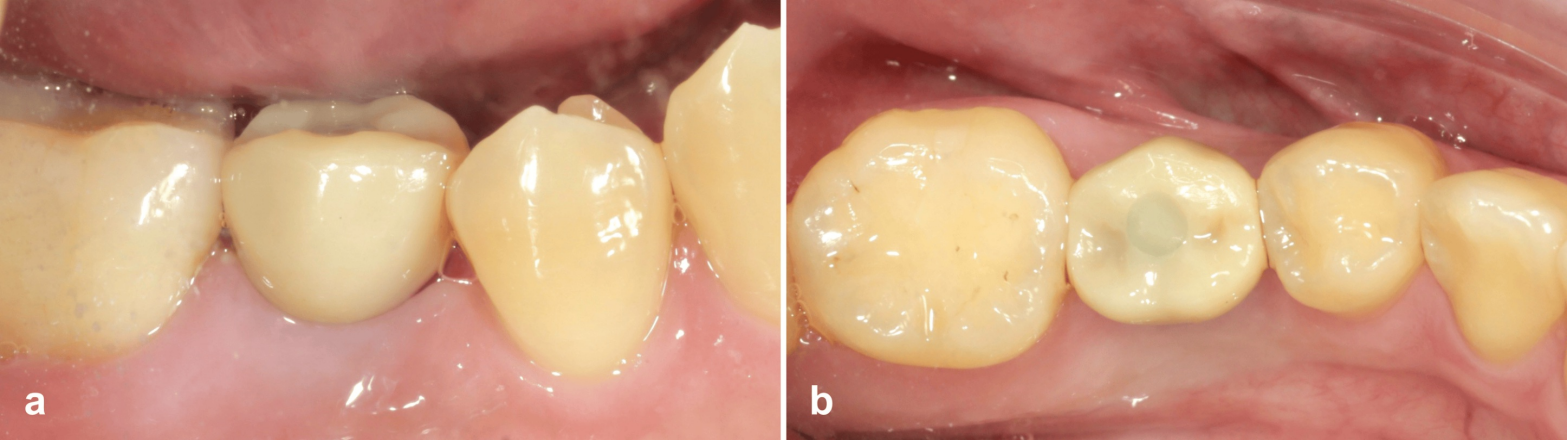
Buccal (a) and occlusal view (b) of the final implant restoration.

**Figure 8:**
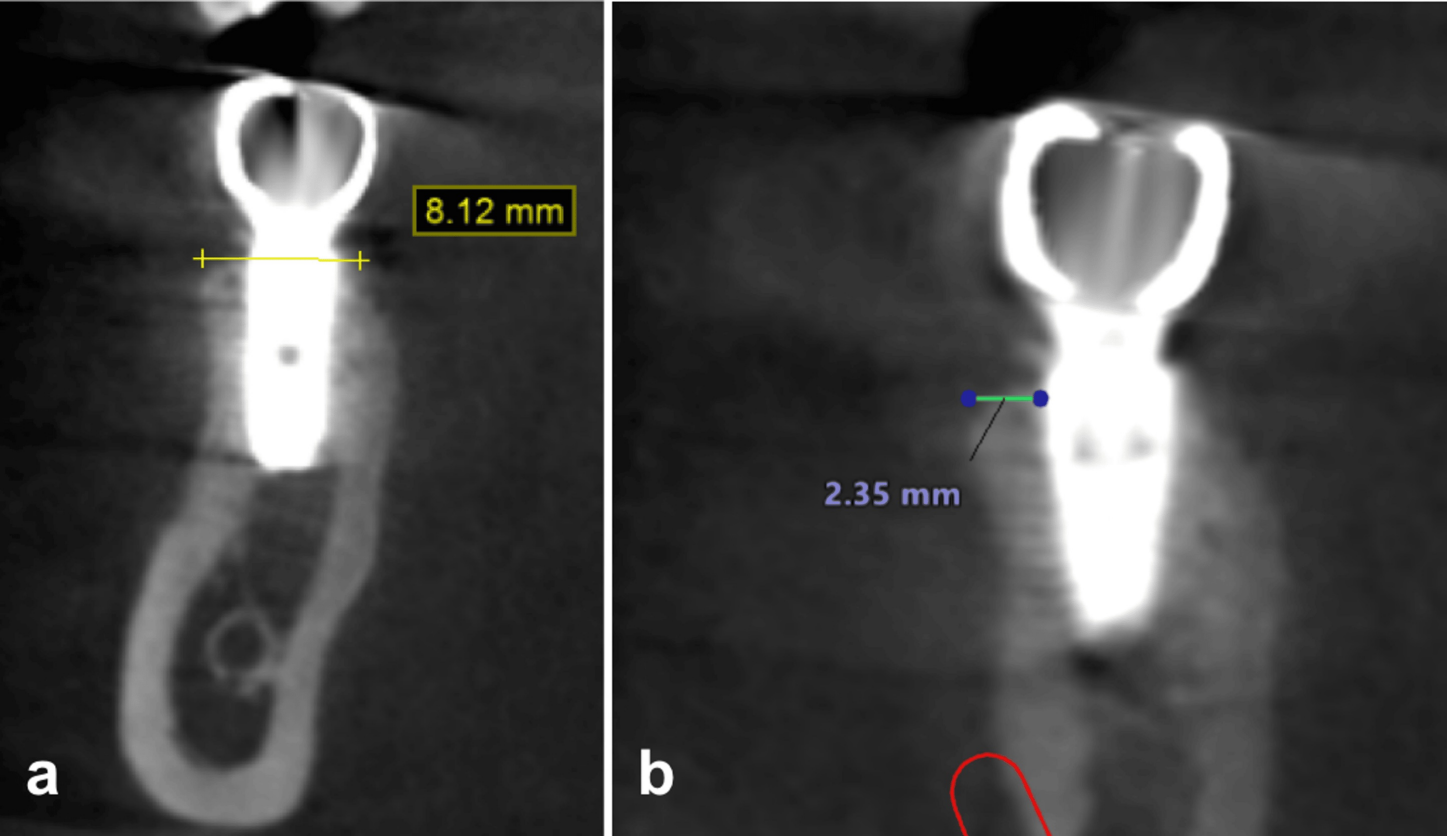
CBCT view of the ridge expansion (a) and the final implant restoration (b). CBCT, cone beam computed tomography

After six months of healing, the healing abutment was removed, and a definitive impression capturing the soft tissue contour was taken using VPS (Aquasil Ultra+, Dentsply Sirona). A custom screw-retained titanium abutment with a zirconia crown was fabricated, followed by occlusal adjustments upon delivery. The keratinized tissue was preserved following the implant restoration (Figure 7). A CBCT scan was retaken a year after implantation, showing a ridge expansion of 8.12 mm, which represents a gain of 1.64 mm in width, alongside a new measurement of buccal wall width of 2.35 mm from the implant surface. A buccal bone wall expansion of 0.22 mm was noted compared to the initial CBCT (Figure 8).

## Discussion

Effective treatment planning is vital for successful implant restoration. It ensures that the implant is accurately positioned and securely placed within sufficient alveolar bone. The ideal placement facilitates the maturation of peri-implant tissues and supports prosthetic restoration [1,2,4].

The 3A-2B rule is a prosthetically driven implant treatment plan that ensures adequate space for restoring the prosthesis while maintaining proper occlusion without violating the biological width, as well as preserving the buccal bone wall [1]. Su et al. [2] recommended that a minimum of 1 mm of buccal bone be retained around the implant and that 2 mm should be explicitly preserved in the anterior region to achieve better esthetic results. Albrektsson et al. [3] determined that marginal bone loss can happen in healthy implants during the first year because of bone remodeling and functional loading. Spray et al. [6] and Monje et al. [7] concluded that a buccal bone wall thickness of greater than 1.8 mm can reduce the chances of complications and contribute to long-term success and implant stability. An inadequate buccal bone wall can jeopardize the integrity of the peri-implant bone, increasing the risk of tissue recession that impacts both aesthetic and functional aspects [7,8].

To enhance the stability of dental implants, it is recommended that sufficient mechanical insertion be ensured in order to attain optimal primary stability during the placement of the implant into the osteotomy socket. Lioubavina-Hack et al. [4] and Monje et al. [5] emphasized the importance of primary stability in achieving successful osseointegration. Throughout the healing process, bone mineralization occurs adjacent to the surface of the implant, facilitating tissue remodeling and enhancing the BIC [4].

In cases of low-density alveolar ridge, the OD method can enhance the osteotomy socket by increasing the density of the lateral bone walls surrounding the implant [13-16]. Huwais and Meyer [14] discovered that trabecular bone compaction occurs during the OD instrumentation, resulting in viscoelastic deformation of the alveolar bone as it recovers from the spring-back effect. This effect allows for implant placement in a narrower osteotomy, enhances the density of the osteotomy and implant primary stability, and promotes faster bone healing through healing chambers at the bone-implant interface. Althobaiti et al. [15] compared OD with conventional osteotomy drilling and found that the ISQ and RFA values were higher in the OD group, achieving better primary stability than traditional dental implant procedures. Kalra et al. [16] concluded that both conventional and OD methods maintained the crestal bone level of the implant. Furthermore, OD exhibited an advantage in achieving higher implant stability during the procedure and the healing stage, enhancing its capacity to improve outcomes and minimize complications of implant therapies.

In addition to improving the primary stability of implants through bone densification, OD preparation also proves effective in facilitating the expansion of residual alveolar ridges. Koutouzis et al. [13] conducted a multicenter retrospective study, concluding that the OD could expand the alveolar ridge width from 1.14 to 2.83 mm based on the original ridge width. The case presentation demonstrated a result of 1.64 mm, which aligns closely with the findings of the study. A randomized controlled trial conducted by Shanmugam et al. [17] has demonstrated the enhancement of implant primary stability achieved through the utilization of OD while preserving the existing bone structures, resulting in ridge expansion and minimizing the likelihood of dehiscence or fenestration. Li et al. [18] conducted a preliminary investigation utilizing OD and conventional osteotomy drilling techniques on bovine rib specimens. Their findings indicated that OD effectively expanded the alveolar ridge with abundant irrigation while preventing bone necrosis due to overheating. Tian et al. [19] investigated the ridge expansion between OD burs and osteotome instrumentation. They concluded that the OD group provides double the value of BIC in comparison to the osteotome group, with approximately 80% of the testing sites anticipating millimeters of ridge expansion. Another clinical study by Salman and Bede [20] also observed ridge expansion in narrow alveolar ridges subsequent to the OD preparation, as well as obtaining a high implant survival rate following the osseointegration.

This case presentation demonstrates the use of OD in an adequate alveolar ridge, resulting in the compression of the buccal bone wall, which facilitates ridge expansion through the spring-back effect, thereby enabling the implant to be positioned with a high insertion torque. The peri-implant tissue and the adjacent gingival tissue are preserved and reshaped without additional regenerative materials due to bone remodeling, which favors the long-term survival of the implant restoration.

## Conclusions

An optimal position for implant placement plays an important role in extending the longevity of the restoration. A treatment plan driven by prosthetic considerations could establish the appropriate depth and angulation for the placement of the implant. Despite the limited number of studies, the OD method for implant placements has been shown to be a reliable technique for achieving primary stability, even within low-density alveolar ridges. Furthermore, OD demonstrates certain outcomes related to alveolar ridge expansion. However, effective use of OD necessitates specifically designed burs and clinical experience with bouncing-pumping motions. Moreover, it remains challenging to determine the increments associated with these outcomes. In cases of insufficient or severe resorption, it is recommended to combine the OD method with additional regenerative procedures for implant placement.
